# Commentary: A mental number line in human newborns

**DOI:** 10.3389/fnhum.2020.00099

**Published:** 2020-03-24

**Authors:** Arianna Felisatti, Jochen Laubrock, Samuel Shaki, Martin H. Fischer

**Affiliations:** ^1^Department of Psychology, University of Potsdam, Potsdam, Germany; ^2^Department of Behavioral Sciences, Ariel University, Ariel, Israel

**Keywords:** spatial-numerical associations, SNARC, mental number line (MNL), spatial frequency (SF), temporal frequency, hemispheric asymmetry, newborns, embodied cognition

Several thousand (Reuters, 2013) studies have investigated why we associate small numbers with left and large numbers with right space. While humans may learn this association through cultural techniques (Zebian, 2005; Shaki et al., 2009; Göbel et al., 2015), its presence in human new-borns (de Hevia et al., 2017) as well as in non-human animals (Rugani et al., 2015; for review Rugani and de Hevia, 2017; McCrink and de Hevia, 2018) requires a biological explanation. Is there an inborn Spatial-Numerical Association (SNA)?

Di Giorgio et al. (2019) provided a positive answer after testing hour-old humans with a habituation paradigm. They exposed neonates to static two-dimensional images depicting 12 black squares. Once the habituation criterion was reached, bilateral test stimuli were exposed. They consisted of identical images displaying a numerosity which was for some neonates smaller (“4”) and for other neonates larger (“36”) than the habituated one. Neonates preferred looking at the left image when tested on 4-square images and at the right image when tested on 36-square images.

These findings imply the presence of SNAs at birth; but covariations of numerosity with non-numerical stimulus features prevented clear conclusions. While previously the number of elements was positively correlated with area, a new experiment implemented a negative correlation between numerosity and area by controlling perimeter. Again two groups of new-borns were tested with a single habituation followed by two lateralized test images: Group one after habituating to a 4-big-square image, preferred looking at the 12-small-square image depicted on their right side; conversely group two habituated to a 36-small-square image, preferentially looked at the 12-big-square image displayed on their left side. Since both groups were tested with the same numerosity (“12”), their different looking preferences indicated that they judged the target in relation to the numerosity and not the area of the habituation pattern. The authors interpreted these findings as evidence for an inborn tendency to map numbers onto space, independent of continuous physical variables.

Vallortigara (2018) suggested that few/many elements, triggering withdrawal/approach behaviors, are associated with negative/positive emotions, preferentially processed by the right/left hemisphere, respectively (Davidson, 2004). Instead, we believe that hemispheric specialization for low-level features (Hellige, 1996; Kauffmann et al., 2014) explains the innate SNAs without directly relying on number concepts. Spatial Frequencies (SFs) are defined as number of dark/light cycles/degree of visual angle. Different spatial frequency ranges represent different information from any visual scene (Goffaux et al., 2005; Flevaris and Robertson, 2016): Low SFs (few cycles/degree) represent few coarse elements, while high SFs (many cycles/degree) represent many detailed elements. Lateralized vertebrates are neuronally specialized for spatial vision (Vallortigara et al., 2011; Rogers, 2017): Behavioral and neuroscientific studies found that when viewing any scene, vertebrates preferentially extract coarse visual features (low SFs) with their right hemisphere and fine details (high SFs) with their left hemisphere (see Figure 1A). This was documented with hierarchical (so-called “Navon”) stimuli (Sergent, 1982; Fink et al., 1996); grating/checkboard patterns (see Figure 1B; Kitterle and Selig, 1991; Martinez et al., 2001; Piazza and Silver, 2014); and natural scenes (Peyrin et al., 2003; Musel et al., 2013). For any visual scene with homogeneous feature distribution, the cross-over of the optic fibers naturally enhances relative smaller numerosities in our left visual field and relative larger numerosities in our right visual field. In human new-borns, their immature inter-hemispheric communication further augments this bias (Salamy, 1978; Deruelle and de Schonen, 1991).

**Figure 1:**
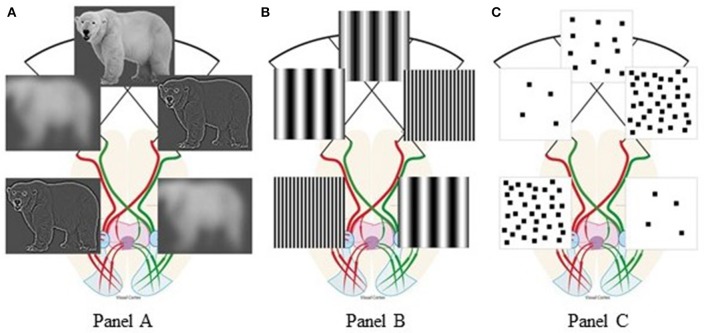
Visual percepts resulting from hemispheric spatial frequency tuning: **(A)** polar bear scenes, adapted from “Figure 3” Panichello et al. (2013), licensed under CC-BY, version 4.0; **(B)** spatial frequency gratings; **(C)** square-pattern stimuli taken from the target article by Di Giorgio et al. (2019). The anatomy of visual pathways is redrawn after “Figure 1. Visual pathway in a primate.” by Larsson (2015), used under CC-BY, version 4.0. Figure 1 is licensed under CC-BY, version 4.0 by Arianna Felisatti.

When we modeled this naturally-occurring visual filtering process on the very stimuli used by Di Giorgio et al. (2019), their behavioral bias emerged from the hemispheric lateralization of SF processing: For any visual scene, patterns with relative few elements preferentially engage the right hemisphere, thus favoring leftward behavior. Conversely, patterns with relative many elements preferentially engage the left hemisphere, thus inducing rightward behavior (Figure 1C). Therefore, when total perimeter but not SF content of the stimuli used to test numerosity effects is experimentally controlled, apparent numerical biases reflect natural lateralization of SF processing. If our SF explanation of Di Giorgio et al. (2019) finding is correct, the resulting association “few-left” and “many-right” holds to the degree that numerosity and SF are correlated, e.g., when large numbers tend to be represented by smaller objects. Although our analysis holds for the above habituation study, the same SF filtering principle applies also to viewing stimuli prior to habituation.

More generally, we suggest that our Brain's Asymmetric Frequency Tuning (BAFT) hypothesis accounts for spatial-numerical associations without further need of cognitive mechanisms. Indeed, it provides evidence not only for the origin of horizontal SNAs, but also for their relative nature: Just as the spatial association of small and large numbers depends on the numerical range (Dehaene et al., 1993), the discrimination between low and high SFs depends on the SF range of a given image (Flevaris et al., 2011; Piazza and Silver, 2017).

The BAFT hypothesis makes predictions for numerical cognition and beyond. We predict: (1) In new-borns, for a given numerosity pattern, spatial associations are driven by its absolute or relative SFs; (2) SNAs driven by SFs generalize across cultures and species; (3) SF selection and, as a consequence, SNAs are different in new-borns predisposed to developing autism (enhanced local processing: Jobs et al., 2018) and dyscalculia (deficit in number acuity: Piazza et al., 2010). Moreover, our hypothesis provides a theoretical framework for SNAs across sensory modalities: Indeed, the new-born's association of few syllables with left-space and many syllables with right-space (de Hevia et al., 2017) might reflect temporal frequency tuning in the auditory cortex. The hemispheric asymmetry would be involved in a second stage, after the attentional system has filtered the relevant frequency (double filtering by frequency; Robertson and Ivry, 2000) or could be intrinsic to the process allowing integration of the signal at different temporal windows (asymmetric sampling in time; Poeppel, 2003; Flinker et al., 2019) from early infancy (Telkemeyer et al., 2009).

In conclusion, nature endows us with specialized brains that impose embodied constraints on how we represent numbers.

## Author Contributions

AF conceived the presented hypothesis. MF and SS contributed to developing the idea. JL helped AF to perform computations on original stimuli used by Di Giorgio et al. (2019) and participated in the discussions. MF supervised all steps of this work. The final commentary reflects the interdisciplinary and international cooperation of AF, JL, SS, and MF.

### Conflict of Interest

The authors declare that the research was conducted in the absence of any commercial or financial relationships that could be construed as a potential conflict of interest.
